# Polyethylene Terephthalate Textiles Enhance the Structural Maturation of Human Induced Pluripotent Stem Cell-Derived Cardiomyocytes

**DOI:** 10.3390/ma12111805

**Published:** 2019-06-03

**Authors:** Mari Pekkanen-Mattila, Martta Häkli, Risto-Pekka Pölönen, Tuomas Mansikkala, Anni Junnila, Elina Talvitie, Janne T Koivisto, Minna Kellomäki, Katriina Aalto-Setälä

**Affiliations:** 1 BioMediTech, Faculty of Medicine and Health Technology, Tampere University, 33140 Tampere, Finland; martta.hakli@tuni.fi (M.H.); risto-pekka.polonen@tuni.fi (R.-P.P.); leo.mansikkala@oulu.fi (T.M.); junnila.anni@gmail.com (A.J.); elina.t.talvitie@gmail.com (E.T.); minna.kellomaki@tuni.fi (M.K.); 2 Microelectronics Research Unit, University of Oulu, FI-90014 Oulu, Finland; janne.koivisto@oulu.fi; 3 Finland and Heart Hospital, Tampere University Hospital, 33100 Tampere, Finland; katriina.aalto-setala@tuni.fi

**Keywords:** textile, PET, biomaterials, iPS-cells, cardiomyocytes, maturation, gene expression

## Abstract

Human-induced pluripotent stem cell-derived cardiomyocytes (hiPSC-CMs) have the potential to serve as a model for human cardiomyocytes. However, hiPSC-CMs are still considered immature. CMs differentiated from hiPSCs more resemble fetal than adult cardiomyocytes. Putative factors enhancing maturation include in vitro culture duration, culture surface topography, and mechanical, chemical, and electrical stimulation. Stem cell-derived cardiomyocytes are traditionally cultured on glass surfaces coated with extracellular matrix derivatives such as gelatin. hiPSC-CMs are flat and round and their sarcomeres are randomly distributed and unorganized. Morphology can be enhanced by culturing cells on surfaces providing topographical cues to the cells. In this study, a textile based-culturing method used to enhance the maturation status of hiPSC-CMs is presented. Gelatin-coated polyethylene terephthalate (PET)-based textiles were used as the culturing surface for hiPSC-CMs and the effects of the textiles on the maturation status of the hiPSC-CMs were assessed. The hiPSC-CMs were characterized by analyzing their morphology, sarcomere organization, expression of cardiac specific genes, and calcium handling. We show that the topographical cues improve the structure of the hiPSC-CMs in vitro. Human iPSC-CMs grown on PET textiles demonstrated improved structural properties such as rod-shape structure and increased sarcomere orientation.

## 1. Introduction

Cardiovascular diseases are the leading cause of death worldwide [1]. Cardiotoxicity is one of the main causes of withdrawal of drugs from the market [2]. Traditionally, new cardiac drugs and the cardiotoxicity of cardiac and non-cardiac drugs have been tested with rodent cardiomyocytes as well as with transfected non-cardiac cells [3,4,5]. However, the results of these experiments are not always applicable to humans. Therefore, more accurate human cardiomyocyte models are needed for preclinical analysis of drugs as well as for basic research and disease modeling of human cardiac diseases [6]. Human-induced pluripotent stem (hiPS) cells can be reprogrammed from any somatic cell by introducing the pluripotency factors [7] and these cells can be differentiated into functional cardiomyocytes with multiple methods, as recently reviewed [8]. However, these cells have been criticized as being immature and more resembling fetal than adult cardiomyocytes (CMs) [9]. Compared to adult human CMs, hiPSC-CMs are small in size, round or multi-angular, and typically single-nucleated, whereas adult CMs are rod-like and large, with 25%–57% of the cells multi-nucleated [10,11]. The aspect ratio can be used as an indicator of the cell shape. Due to the round shape, hiPSC-CMs have an aspect ratio of (2–3): 1, whereas adult CMs are clearly longitudinally-oriented with an aspect ratio of (5–9):1. Sarcomeres of the hiPSC-CMs are disorganized and short (<2 μm), the sarcoplasmic reticulum is poorly developed, and the sarcolemma exhibits no transverse tubules [10,12]. The electrophysiological properties and the gene expression of the hiPSC-CMs differ from adult CMs [12].

The contractile ability of CMs is enabled by multiple sarcomere units that are integrated in the cytoskeleton of the cell [13]. The efficiency of the CM contraction relies on the proper orientation and length of the sarcomeres and on the rod-like shape of the cell [14]. Thus, the structural maturation of the cells has been the focus when studying maturation methods for hiPSC-CMs. Multiple methods and strategies have been suggested to improve the maturation of hiPSC-CMs in vitro, including topographical cues, substrate stiffness, medium additives, mechanical and electrical stimulation, genetic manipulation, and co-culture with other cell types [10,15]. However, a deeper understanding of the maturation process of hiPSC-CMs is still required to develop platforms to promote the maturation of the cells and producing hiPSC-CMs more resembling adult CMs.

Various scaffolds have been studied to orient hiPS-CMs, such as electrospun-aligned fiber textiles [16,17] and micro-grooved culture substrates [18]. Most of the cell cultures in vitro are coated on flat surfaces, which provide a two-dimensional (2D) environment for the cells. 2D culture forces cell polarization by providing cell-extra cellular matrix (ECM) connections on only one side of the cells [19,20]. Cells in 2D are usually flat because they try to spread out on the surface. By providing a three-dimensional (3D) environment with proper topographical cues and an extracellular matrix, cells can create more cell–ECM connections, which potentially affect cell proliferation and even differentiation and maturation [20]. Stiffness of a flat, 2D culture substrate affects cell properties, proliferation, and differentiation [21]. To increase the cardiac functionality and maturity, hiPS-CMs have been cultured with, for example, endothelial cells, and this has shown to enhance cardiomyocyte proliferation and functionality [22]. Co-culture of hiPS-CMs with cardiac fibroblasts has improved the structural and functional properties of the cells [23,24]. A similar maturation-enhancing effect was observed when cardiomyocytes were cultured on top of the vascular-like network produced from endothelial cells and fibroblasts [25,26]. Stem-cell-derived cardiomyocytes aligned according to the vascular structures of the network and their sarcomere structures were more oriented. 

Textiles create a 3D culture environment and provide topographical support for different types of cells. Having a highly interconnective porous structure, textiles enable access of media and nutrients to the cells inside the material. Weaving is a conventional and basic textile technique that can also be used to fabricate tissue engineering scaffolds. Weaving enables the formation of textile structures with controllable properties, such as porosity, orientation, morphology, and mechanical properties. These parameters can be modified, for example, by changing the number of filaments, filament diameter, and weaving patterns. Biostable polyethylene terephthalate (PET) is one of the most used polyesters, and has many applications including in biomedical applications, for example as hernia meshes. It has also been used in many cell culture studies. The raw material of fibers can be changed too, and in the future, biodegradable textiles could be used as a vehicle for implantation of cardiomyocyte sheets for myocardial ischemia or scar repair applications [27,28,29]. 

In this study, PET textiles were used as culture substrates for hiPSC-CMs. The aligned textile fibers were hypothesized to provide sufficient topographical cues to improve the maturation state of hiPSC-CMs. The PET textiles had different weaving patterns, including a plain weave and a plain weave derivative, which altered their topography and other properties. They were coated with different biological compounds: Geltrex^TM^ (Thermo Fisher Scientific) and gelatin were used. The CMs were characterized by cell morphology, sarcomere organization, expression of cardiac specific genes, and calcium handling properties.

## 2. Materials and Methods 

### 2.1. l Polyethylene Terephthalate Textile

**Figure 1:**
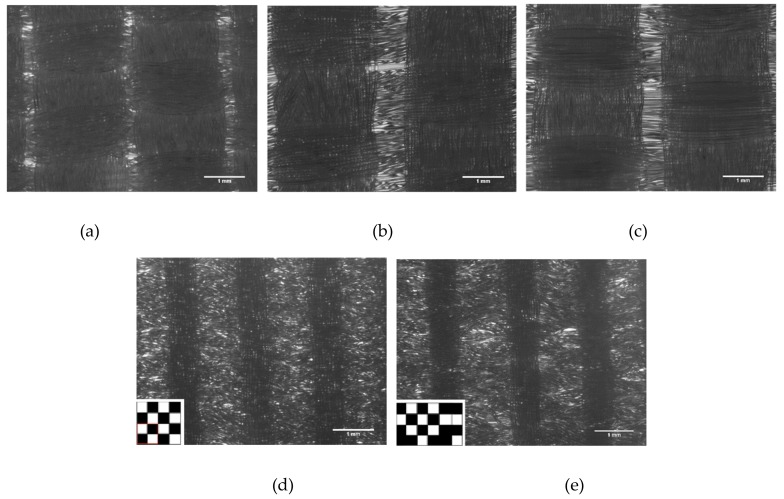
Structures of the polyethylene terephthalate (PET)-based textiles types 1–5 used in the present study, imaged with a Zeiss Axio Vert.A1 microscope (bright field) and AxioCam MRc5 camera using 5× objective. PET types 1–3 (**A**–**C**, respectively) were commercial textiles produced by Inka Oy, Killinkoski, Finland. PET types 4 and 5 (**D**,**E**, respectively) were produced at Tampere University, Tampere, Finland. Warp beams were provided by Finn-Nauha Oy, Haapamäki, Finland (yarn from Sinterama, Biella, Italy). The weaving type of the type 4 and 5 PET textiles were plain weave and plain weave derivative, respectively, as shown in lower left corner of the images.

Five different PET textiles were used (PET 1–5, Figure 1). They differed in color, texture of the fibers used as warp and weft, single filament (fiber) thickness, textile density, and pattern of the textile according to the details listed in Table 1. The average single filament thickness (measured from immunostaining images using autofluorescence of the fibers and ImageJ software used in the textiles varied between 20.4 and 24.4 µm without significant differences. All the textiles were of narrow fabric type, i.e., they were woven by a narrow-weaving loom having aligned fibers in their structure as warps. Perpendicular to the warps, the interweaving wefts formed the structure for the textile according to the pattern followed. The textile pattern of PET 1–4 was the same, plain weave, but other parameters varied. PET 5 was an in-house-designed plain weave derivative (pattern drawings in Figure 1). The textiles were woven narrow fabrics and the width of the textiles was 9 mm. The textiles were cut to pieces of 7–8 mm before final sterilization and cell seeding. Textiles were washed with ethanol (3–4 times washing with excess amounts of alcohol), followed by thorough drying before heat treatment. All the textiles were heat treated to stabilize the textile structure for the cell culture experiments.

### 2.2. Textile Coating

The PET textiles were coated to create a thin layer on the textile to increase attachment without compromising textile topography. Five different surface coatings were tested in the optimization phase of the present study: Geltrex^TM^, Gelatin, dopamine-bound gelatin coating, plasma-treatment of the textile without any coating, and plasma-treatment and gelatin-coating. Two replicates of each coating were used in each experiment. The glass coverslips were used as the control surface for hiPS-CMs. During the optimization phase of the experiment, all five PET types were used, and all different coating methods were tested for each PET type. In the experiment phase, only the gelatin coating was used for PET type 5.

To improve the attachment of the coating, as well as the attachment of the cells, PET textile was plasma-treated prior to gelatin coating. The plasma treatment was performed with plasma system Pico, Model 2, standard system controlled via PC and Windows CE operating system (control type C: PCCE control) and with reactive ion etching electrode. The electrode was type E (stainless steel), the generator was type D (13.56 MHz, 0-100W) (Diener electronic GmbH, Ebhausen, Germany) and the vacuum pump was Leybold 19 SC5D (Leybold Vacuum GmbH, Cologne, Germany). The gas used in the plasma treatment was O_2_ and PET textiles were treated for 2 min in 0.4 mbar pressure with 50 W. 

Prior to coating, the textiles and the coverslips were disinfected by washing with 70% ethanol (Altia, Rajamäki, Finland) and left to dry properly (1–2 h) before coating in the laminar hood.

Geltrex^TM^ and gelatin were used as coating materials. Geltrex^TM^ (Thermo Fisher Scientific, Waltham, Massachusetts, USA) was thawed and diluted 1:100 in KnockOut Dulbecco’s Modified Eagle Medium (DMEM) (Thermo Fisher Scientific, Waltham, Massachusetts, USA). We pipetted 500 µL and 150 µL of diluted Geltrex^TM^ on PET textiles and coverslips, respectively, which were incubated at 37 °C for 1 hour. Excess coating was aspirated just prior to the cell plating in all cases. 

Gelatin coating was performed in three different ways. In the first method, Type A porcine gelatin (Sigma-Aldrich, Saint Louis, Missouri, USA) was dissolved in phosphate buffered saline (PBS) to form 0.1% solution. We pipetted 500 µL and 150 µL of 0.1% gelatin solution on PET textiles and coverslips, respectively, which were incubated in room temperature for an hour. The second method involved using plasma treatment before gelatin coating. The third method was used to improve the attachment of gelatin to a polymer [27]. Dopamine hydrochloride (Sigma-Aldrich, Saint Louis, Missouri, USA) was used to crosslink gelatin with the PET fibers. The PET fibers were incubated in 2 g/L dopamine solution for 24 h on a shaking bed at room temperature. After washing with distilled water, the samples were incubated in 5% (w/v) Gelatin type A (Sigma-Aldrich, Saint Louis, Missouri, USA) solution for 24 h at 37 °C. After incubation in gelatin, the samples were washed overnight in distilled water at 37 °C to remove non-chemically bound gelatin. 

### 2.3. Cell Culture and Differentiation of hiPSC-CMs

The hiPSC line UTA.04602, produced from dermal fibroblasts of a healthy individual and cultured as previously described [30], was used in the study. The ethical committee of Pirkanmaa Hospital District (Tampere, Finland) approved collection of biopsies for generating patient-specific hiPSC lines and written informed consent was obtained from all the donors (Aalto-Setälä R08070). The hiPSCs were cultured in mTeSR1 medium (STEMCELL Technologies, Vancouver, Canada) on a Geltrex^TM^ (Thermo Fisher Scientific, Waltham, Massachusetts, USA)-coated surface. The culture medium was changed three times a week for the cells and they were passaged for a one-week culture using Versene (Thermo Fisher Scientific, Cibco, Billings, Montana, USA).

Small molecule differentiation was achieved as previously described [31] with small exceptions. In short, the differentiation was initiated when the hiPSC-culture was 100% confluent (day 0) by changing the mTeSR1 medium to insulin-free RPMI/B27 (Thermo Fisher Scientific, Cibco, Billings, Montana, USA) medium containing 8 µM CHIR99021 (Tebubio, BPS Bioscience, San Diego, California, USA) and 0.5% penicillin/streptomycin. After 24 hours, the medium was changed to fresh insulin-free RPMI/B27 medium. On day three, half the medium was collected from the wells and mixed with fresh insulin-free RPMI/B27 medium. IWP-4 (Tocris, Bristol, England) was mixed with the medium so that the final concentration was 5 µM. The rest of the old medium was exchanged to IWP-4-containing medium. On days five and seven, the medium was changed to fresh insulin-free RPMI/B27 medium, and from day 10 forward, half the medium was changed three times a week to fresh RPMI/B27 medium with insulin (Thermo Fisher Scientific, Cibco, Billings, Montana, USA).

### 2.4. hiPS-CM Dissociation and Magnetic-Activated Cell Sorting 

hiPSC-CMs were dissociated using two methods. For the PET coating optimization phase, the hiPS-CMs were dissociated using Collagenase A and suspended into a suspension medium containing KnockOut DMEM with 10% fetal bovine serum (Biosera, Nuaille, France), 1% non-essential amino acids (NEAA), 1% GlutaMAX-I (100×) (all from Thermo Fisher Scientific, Cibco, Billings, Montana, USA), and 0.5% penicillin/streptomycin (Lonza, Basel, Switzerland) [32]. 

To improve the purity of the hiPSC-CM population in the following experiments with PET 5, the cardiomyocytes were dissociated and separated from other cell types using magnetic-activated cell sorting (MACS) on day 21–27 of the differentiation. The cells were dissociated using a Multi Tissue Dissection Kit 3 (Miltenyi Biotec, Bergisch Gladbach, Germany) following the manufacturer’s instructions. MACS sorting was performed using PSC-Derived Cardiomyocyte Isolation Kit, human (Miltenyi Biotec, Bergisch Gladbach, Germany). After cell sorting, the cells were resuspended in the suspension medium described above and the cells were plated on the gelatin-coated PET 5 textiles and gelatin-coated glass coverslips, which were used as controls.

### 2.5. Calcium Imaging

Calcium imaging was performed on day 12 after plating the cells to the PET 5 textiles. Ten independent PET 5 samples and two control samples were analyzed. Imaging was performed as previously described [33]. Shortly, the cells were loaded with 4 µM Fluo 4 AM (Thermo Fisher Scientific, Waltham, Massachusetts, USA) for 30 minutes at 37 °C. The sample was placed into an imaging chamber (RC-25, Warner Instruments, Hamden, Connecticut, USA) and the chamber was placed onto an Olympus XI71 microscope (Olympus, Tokyo, Japan) and connected to a perfusion system. Cells were perfused with 37 °C pre-heated perfusate solution consisting of 137 mM NaCl, 5 mM KCl, 1.2 mM MgCl_2_, 0.44 mM KH_2_PO_4_, 4.2 mM NaHCO_3_, 2 mM CaCl_2_, 1 mM Na pyruvate, 5 mM D-glucose, and 20 mM HEPES dissolved in distilled water (pH adjusted to 7.4 with NaOH).

The adrenaline response of CMs on PET 5 was evaluated with 1 µM adrenaline (Sigma Aldrich, Saint Louis, Missouri, USA) from six independent samples. Baseline was recorded and hiPSC-CMs were treated with adrenaline for one minute and their response was recorded. Before a new baseline measurement, the adrenaline was washed off for at least two minutes. The recordings were performed with an Olympus XI71 microscope (Olympus, Tokyo, Japan) using ANDOR iXon+ camera and an Olympus UApo 20× 0.75 NA air objective and Live Acquisition software (TILL Photonics, Munich, Germany).

For calcium imaging analysis, single-beating hiPSC-CMs were selected as regions of interest and the analysis of fluorescence (ΔF/F_0_) videos were recorded using TILL Photonics Offline Analysis. The clampfit data analysis module of Axon pClamp 10 Electrophysiology Data Acquisition & Analysis software was used for peak detection (Molecular Devices, San Jose, California, USA). The studied peak parameters included peak duration, rise time from 10% to 90%, decay time from 90% to 10%, and peak frequency.

### 2.6. Immunocytochemistry

Immunocytochemistry was performed on day 10–11 after plating the cells on the PET 5 textile samples. The samples were fixed with 4% paraformaldehyde, blocked with 10% normal donkey serum (Biowest, Nuaille, France) solution, and stained with goat anti-cardiac troponin T (1:1,000, Abcam) and mouse anti-MyBPC3 (1:400, Santa Cruz Biotechnology, Dallas, Texas, USA) at 4 °C overnight. Donkey anti-goat Alexa Fluor 568 and donkey anti-mouse Alexa Fluor 488 (1:800, Thermo Fisher Scientific, Waltham, Massachusetts, USA) were used as secondary antibodies. The cell nuclei were stained using Vectashield mounting medium with DAPI (Vector Laboratories, Burlingame, California, USA). Fluorescence was visualized with a Nikon A1R+ Laser Scanning Confocal Microscope (Nikon, Tokyo, Japan) using a Nikon Apo 60× 1.40NA oil objective and with Zeiss Axio Imager.M2 with ApoTome.2 and AxioCamHRm3 camera using a Zeiss EC Plan-Neofluar 40× 1.30NA oil objective.

### 2.7. Analysis of Cell Alignment and Sarcomere Orientation

The orientation and sarcomere length of the hiPSC-CMs cultured on the PET textiles were analyzed from microscopy images using a spectral analysis tool, CytoSpectre [34]. CytoSpectre allows quantification of orientation and size distribution of cellular structures by using Fourier transform. In this study, the circular variance and wavelength of the detailed spectral component were used to determine the sarcomere orientation and modal sarcomere length of the hiPSC-CMs, respectively. Circular variance ranges from zero to one, with zero describing perfect anisotropy and one describing perfect isotropy. CytoSpectre determines the axes of the cell, which can be used to determine the aspect ratio. Prior to the analysis, the images were processed with ImageJ for masking.

### 2.8. Quantitative Reverse Transcription-Polymerase Chain Reaction (qRT-PCR)

hiPSC-CMs were prepared for qRT-PCR on day 1 and day 11 after plating the cells as previously described [35] to study the expression of several cardiac related genes. PET 5 and control samples (cells from glass coverslips) were collected from six independent experiments (*n* = 6). Two replicate samples from each independent experiment were collected. The cells were lysed with lysis solution of a CellsDirect One-Step qRT-PCR Kit (Invitrogen, Carlsbad, California, USA) following the manufacturer’s protocol. The lysis was stored at −80 °C until genomic DNA degradation with DNase I and reverse transcription-specific target amplification (RT-STA) using the CellsDirect One-Step qRT-PCR Kit. Biomark HD (Fluidigm Corporation, San Francisco, California, USA) was used to perform the real-time qPCR according to the manufacturer’s protocol. All samples were run as duplicates in Fluidigm Dynamic array-plates and the 2-ΔΔCT [36] method was used to calculate relative expression. TATA-box binding protein (*TBP*), eukaryotic translation elongation factor 1 alpha 1 (*EEF1A1*), and glyceraldehyde-3-phosphate dehydrogenase (*GAPDH*) were used as endogenous control genes for data normalization. In assessment of the relative expression, day one samples were used as a calibrator for the data. These samples were similar to the controls samples, the cells were plated to glass coverslips, but cells were lysed one day after plating. Cells were collected from four coverslips (*n* = 4). The TaqMan assays used are listed in Table 2.

### 2.9. Statistical Analysis

The statistical significance of the differences in circular variance, sarcomere length, and height to width ratio of the hiPSC-CMs was assessed by Mann–Whitney U test where *p* < 0.05 was considered statistically significant. Assessing the statistical significance of differences in gene expression levels was performed using the Kruskal–Wallis test with Bonferroni correction. When comparing the calcium baseline measurements to adrenaline measurements, related samples’ Wilcoxon Signed Rank Test was used. *p* < 0.05 was considered statistically significant. The data are presented as mean ± standard deviation.

## 3. Results

### 3.1. Attachment of the hiPSC-CMs to the PET Textiles

Five PET textiles (Figure 1) were tested as a scaffold for the hiPS-CMs. None of the fiber-related parameters (thickness range of the fibers and straight vs. textured quality of the fibers) or the weave pattern changed the behavior of the cells, but all the studied PETs (1–5) supported the growth of the hiPS-CMs in a similar manner (data not shown). PET 5, with a plain weave derivative pattern, was chosen for the following experiments. A combination of plain weave derivative pattern and the reed density used produced the most variating topography for the studied PET textile samples (Figure 1). PET 5 was also blue and had slight autofluorescence, which made the fibers visible with fluorescent imaging. 

Gelatin has been used as a basic coating material for hiPS-CMs culturing in our laboratory; therefore, it was used also in the above-mentioned PET textile screening study. However, the number of the attached hiPS-CMs remained relatively low. To improve the cell attachment on the PET textile, coating with commercial basement membrane matrix Geltrex^TM^ was also tested. In addition, plasma treatment prior to gelatin coating and dopamine-bound gelatin were tested. There were no clear differences in the cell attachment (Figure S1) or structural maturation state of the hiPS-CMs (Table S1) with the coating material or plasma treatment. Thus, after testing multiple PET textile types and coatings, PET 5 with normal gelatin coating was chosen for further experiments. 

### 3.2. hiPSC-CM Morphology, Sarcomere Orientation, and Sarcomere Length

**Figure 2:**
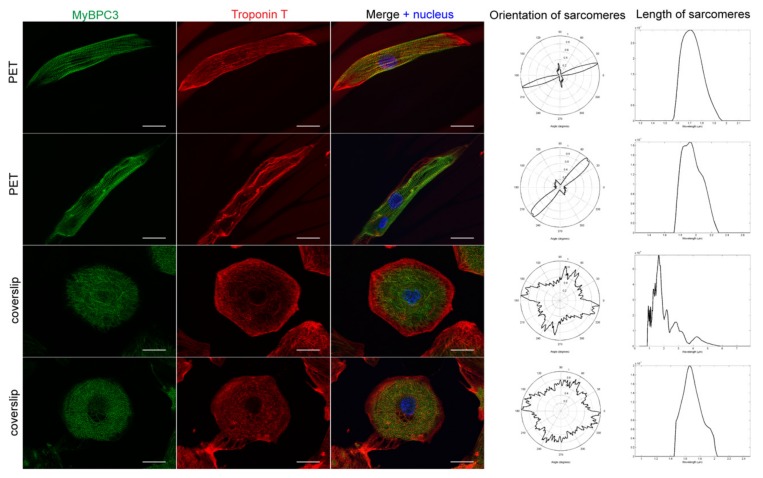
Two representative examples of the structure of human-induced pluripotent stem cell- derived cardiomyocytes (hiPSC-CMs) cultured on gelatin-coated polyethylene terephthalate (PET)-based textiles (PET) textiles and coverslips (controls). The hiPS-CMs were immunostained with myosin binding protein C (MyBPC3) (green) and Troponin T (red). The nuclei of the cells were stained with DAPI (blue). Scale bar is 25 µm. On PET 5, the cells and their sarcomeres clearly aligned according to the fibers of the textile, whereas the control cells exhibited no longitudinal axis or sarcomere orientation to one direction. Orientations of the sarcomeres were analyzed with CytoSpectre. The analysis results of sarcomere orientation and length of sarcomeres confirmed that the orientation of the sarcomeres improved when the cells were cultured on PET 5 textiles, but the sarcomere length distribution in the cells did not differ significantly.

**Figure 3:**
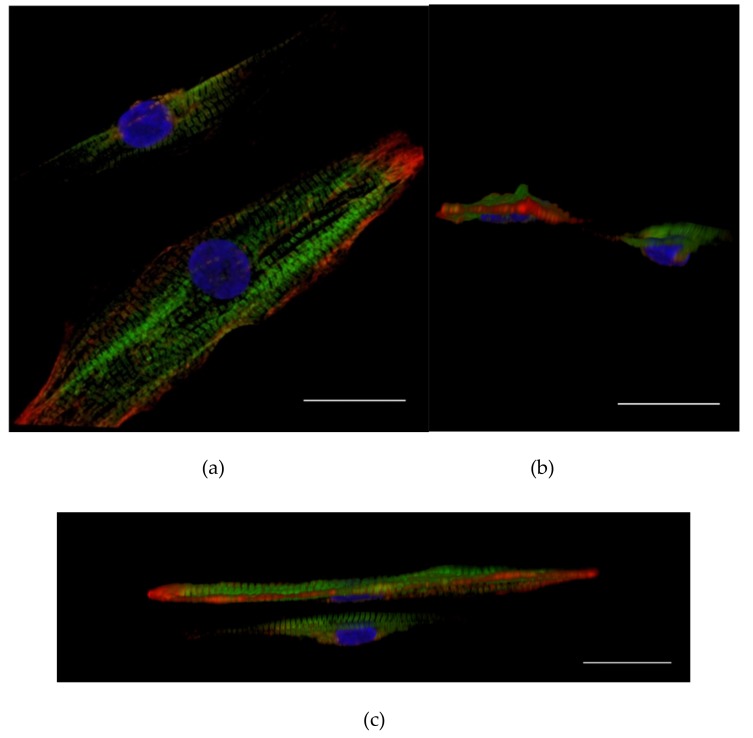
(**a**–**c**) Confocal images from single human induced pluripotent stem cell-derived cardiomyocytes (hiPSC-CMs) on the polyethylene terephthalate PET type 5 textiles, with Troponin T (red), myosin binding protein C, MYBPC (green), nuclear stain DAPI (blue) from different projections. Images reveal that the hiPS-CMs were aligned with the PET fibers. However, the cells wrapped around the single PET 5 fibers and exhibited a relatively flat structure. The sarcomeres of the cells were clearly oriented along the fibers. Scale bar is 25 µm.

hiPSC-CMs cultured on PET 5 and glass coverslips were immunolabeled with Troponin T and Myocin binding protein C3 (MyBPC3) antibodies. Qualitative analysis revealed that the cells aligned according to the PET 5 textile fibers and exhibited clearly elongated structures and increased sarcomere orientation, as shown in Figure 2. The orientation of the CM sarcomeres was significantly higher on PET 5 (*n* = 98) compared to controls (*n* = 174), which was indicated by the lower circular variance (0.611 ± 0.162 and 0.882 ± 0.069, respectively; *p* < 0.05). Table 3 shows examples of the distribution of the sarcomeres in hiPSC-CM cultured on PET 5 and coverslip. Sarcomeres in CMs grown on PET 5 were more oriented than those in the controls. The difference in sarcomere length (Table 3) between PET 5 and control samples was not significant and was 1.736 ± 0.187 µm and 1.749 ± 0.122 µm on average, respectively. The shape of CMs was determined using the aspect ratio, and CMs grown on PET 5 had significantly higher aspect ratios than controls (4.915 ± 2.263 and 1.567 ± 0.455, respectively; *p* < 0.05), indicating that the cells exhibited a more rod-like structure essential for efficient contraction (Figure 2). However, confocal imaging revealed that the hiPS-CMs are still flat and wrap around single PET fibers (Figure 3).

### 3.3. Calcium Handling

Differences in the calcium handling properties between hiPSC-CMs cultured on PET 5 textiles and coverslips were analyzed after 12 days of culture (the age of the cells was 33–39 days after initiation of differentiation). Cells exhibiting normal calcium transients were distinguished from those exhibiting arrhythmias and analyzed separately. The structure of the PET 5 did not hinder the Ca^2+^ imaging. There was no significant difference in the Ca^2+^ peak duration between CMs cultured on PET 5 (*n* = 160) or the control (*n* = 40) plates (582 ± 229 ms and 590 ± 202 ms, respectively; Table 4). However, there were statistically significant differences in rise and decay times, which were 112 ± 49 ms and 295 ± 131 for PET 5 samples and 90 ± 41 ms and 324 ± 96 ms for control samples (*p* < 0.05), respectively. This indicates that the release of calcium was slower while the uptake of calcium was faster for CMs grown on PET 5 compared to the control. Additionally, the amplitude of the peaks was significantly lower in CMs grown on PET 5 compared to controls (0.048 ± 0.037 ΔF/F_0_ and 0.067 ± 0.030 ΔF/F_0_ for PET and control samples, respectively; *p* < 0.05), indicating that control cells released more calcium during contraction cycles. The beating frequency was significantly higher in CMs grown on PET 5 compared to controls (0.93 ± 0.52 Hz and 0.75 ± 0.34 Hz, respectively; *p* < 0.05).

**Figure 4:**
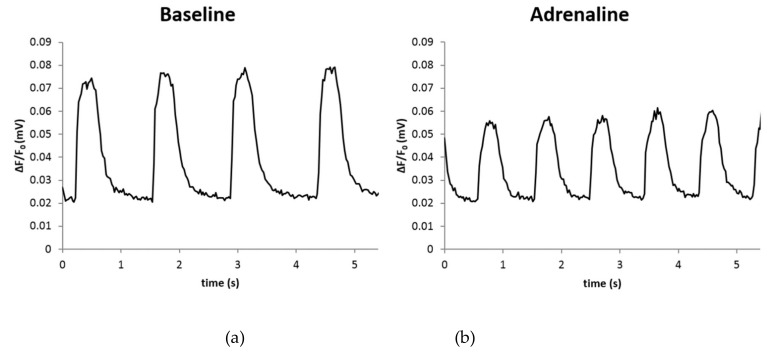
Adrenaline significantly increased beating frequency and decreased peak duration (**a**) when compared to the baseline (**b**) in hiPS-CMs grown on PET 5 textiles.

The response of hiPS-CMs cultured on PET 5 to adrenaline and its effect on calcium handling properties was studied (*n* = 43) (Table 5 and Figure 4). There were statistically significant differences in the peak parameters between baseline and adrenaline measurements (*p* < 0.05). The peak duration decreased by 4.8% (648 ± 101 ms at baseline vs. 614 ± 87 after adrenaline). The peak amplitude decreased by 61.4% (0.0360 ± 0.0183 ΔF/F_0_ at baseline vs. 0.0310 ± 0.0139 ΔF/F_0_ after adrenaline). The rise time increased by 6.1% (115 ± 31 ms at baseline vs. 122 ± 34 ms after adrenaline). The decay time decreased by 7.6% (328 ± 73 ms at baseline vs. 303 ± 63 ms after adrenaline). The beating frequency increased by 21.2% (0.709 ± 0.254 Hz at baseline vs. 0.859 ± 0.242 Hz after adrenaline) as expected. 

### 3.4. Expression of Cardiac-Specific Genes

**Figure 5:**
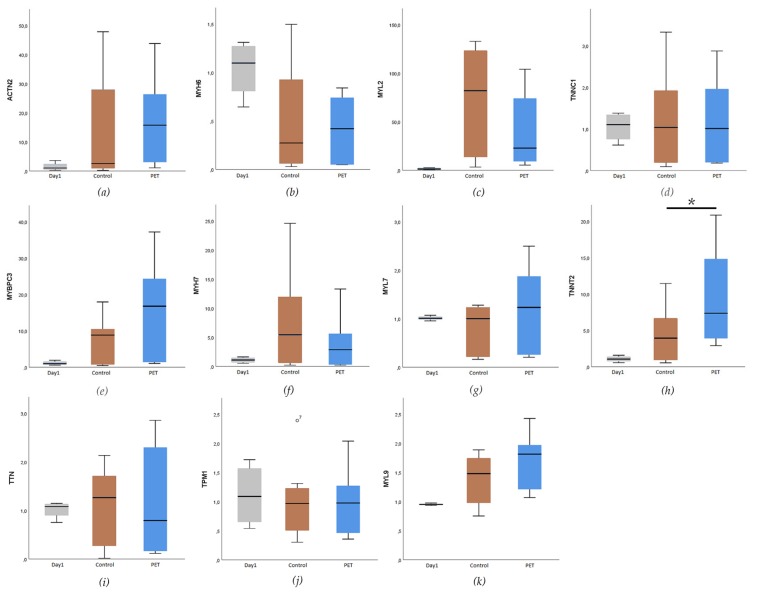
Expression levels of genes coding for the cardiac specific structural proteins: (**a**) α-actinin 2 (*ACTN2*), (**b**) myosin heavy chain 6 (*MYH6*), (**c**) myosin regulatory light chain 2 (*MYL2*), (**d**) slow skeletal and cardiac type troponin C1 (*TNNC1*), (**e**) myosin binding protein C, cardiac (*MYBPC3*), (**f**) myosin heavy chain 7 (*MYH7*), (**g**) myosin regulatory light chain 7 (*MYL7)*, (**h**) cardiac type troponin T2 (*TNNT2*), (**i**) titin (*TTN*), (**j**) tropomyosin (*TPM1*), and (**k**) myosin regulatory light chain 9 (*MYL9*). Only the expression level of (h) *TNNT2* was significantly higher in hiPSC-CMs cultured on gelatin coated PET 5 textiles compared to the control sample (*p* < 0.05), marked with *.

The expression of cardiac specific genes was analyzed using qRT-PCR. Data from PET 5 (*n* = 6) and control coverslip (*n* = 6) samples collected at day 11 (the age of the cells was 32–38 days after differentiation initiation) were calibrated with the day 1 (*n* = 4) samples. Two biological replicates were analyzed from each sample, and all the samples were run as triplicates. TATA-box binding protein (*TBP*), eukaryotic translation elongation factor 1 alpha 1 (*EEF1A1*), and glyceraldehyde-3-phosphate dehydrogenase (*GAPDH*) were used as endogenous control genes for normalization. Overall, high variation was observed between the experiments. The expression levels of the genes coding for the contractile proteins, such as Troponin T (*TNNT2*), myosin binding protein C (*MYBPC*), and cardiac alpha actinin (*ACTN2*) had an increasing trend after the 11 days of culturing on both the PET 5 and control hiPS-CMs. However, only expression of TNNT2 was significantly higher in the hiPSC-CMs cultured on PET 5 compared to controls on glass coverslips (*p* < 0.05, Figure 5). The expression levels of the genes coding for cardiac ion channels were similar for the CMs cultured on glass coverslips and PET 5 (Figure S2). 

## 4. Discussion

Several studies have reported the positive effects of a structured substrate on the maturation of hiPSC-CMs [16,17,18,37,38,39]. These findings have suggested that topographical cues can align the hiPSC-CMs and improve their morphology. In this study, we assessed the possibilities of textile structures being used as a culturing scaffold for hiPSC-CMs. The differentiated hiPSC-CMs were cultured on textile constructs, and their structure, sarcomere orientation, cardiac function, as well as expression of cardiac specific genes were analyzed. Based on our results, culturing hiPSC-CMs on PET textiles improved their structural properties, such as elongation and sarcomere orientation, as well as improved the expression of sarcomeric genes such as *TNNT2*. 

Textiles as scaffolds have beneficial properties for cell culture experiments. First, they provide topographical cues for the cells and enable the transportation of nutrients through the porous material. Secondly, textiles provide support to form tissue-like three-dimensional structures by superimposing multiple textile layers with one or multiple types of cells. In the present study, PET-based textiles were chosen as the scaffold for hiPSC-derived CMs, since PET is one of the most used polyesters in biomedical applications [40].

The hiPSC-CMs cultured on the PET textile were clearly elongated along the fibers of the textile and the sarcomeres were more aligned than in CMs cultured on the standard gelatin coated glass coverslips. A similar alignment of stem cell-derived CMs and their sarcomeric structures was reported when cultured on micro-grooved substrates [18]. Compared to the micro-grooved cultures, PET culturing had a similar effect on the hiPS-CMs even though the cells were wrapped around the fibers of PET textiles, and therefore exhibited rather flat and rounded shapes compared to the more 3D structure of the CMs cultured in the microgrooves [18]. Rao et al. suggested that the alignment of the sarcomeric structures and the orientation of the cell along the fibers are enhanced because the focal adhesion complexes of the cells are formed parallel to the microgrooves. Therefore, the contraction of the cell is directed along the grooves and this orients the sarcomeres as well as the whole cell along the grooves. Similar phenomena might cause the orientation of hiPSC-CMs on PET fibers. The hiPS-CMs focal adhesion complexes might be formed parallel to the fibers, and due to this, the sarcomeres are oriented toward the same direction as the fibers. In addition to the microgrooves, nanogrooves have been reported to facilitate the alignment of cardiomyocytes [41]. Both of these studies speculated that the edges of the grooves are especially crucial in the formation of the focal adhesion complexes. Our data also showed that single 22.0 ± 1.4-µm-diameter fiber without any edges had a similar orienteering effect on the hiPS-CMs. 

Culturing of hiPS-CMs on PET textile did not have an effect on the sarcomere length, which was approximately 1.7 µm and still shorter compared to the average of 2.2 µm in primary adult human CMs [41]. The sarcomere lengths reported for stem cell-derived cardiomyocytes have ranged from 1.4–1.7 µm [42] and some studies have reported that structured culture substrates that provide topographical cues have increased the sarcomere length. However, the sarcomere length has not reached the length of primary CMs [16,39,43]. An elongated structure and sarcomere orientation are important in terms of the efficiency of the CM contraction as the magnitude the CMs can contract in one direction increases with increased elongation and sarcomere orientation [14].

As mentioned above, topographical cues have been shown to align and improve the morphology of hiPSC-CMs toward a more mature phenotype. However, their effects on functionality and gene expression of cardiac specific genes have been controversial. In the present study, PET culturing enhanced the expression of the sarcomeric gene *TNNT2* compared to the hiPSC-CMs cultured on a flat surface. No statistically significant changes were observed in the expression of other cardiac-specific structural genes or in the genes encoding cardiac ion channels. Similar results have been reported earlier; topographical cues have oriented the cells but there has been no significant improvement at the gene expression level [18,43]. However, culturing of iPS-CMs on electrospun fibers had positive effects on the gene expression levels of genes coding for cardiac structural proteins as well as ion channels when compared to culturing on cell culture plastic. Interestingly, positive effects were seen observed regardless of the alignment of the electrospun fibers [16]. The time scale in these studies had been similar, cells were cultured on the scaffolds for two weeks. The longer culturing time of the hiPS-CMs on the surfaces providing topographical cues might enhance the expression level of cardiac cardiac-specific genes in addition to the structural maturation. Furthermore, the controversy in gene expression studies might be due to the other cell types present in the cultures. Even though hiPS-CMs were sorted in the present study, there are other cell types left remained in the cultures with in varying quantities. Due to the lack of cardiac cardiac-specific markers for normalization, this phenomenon can cause the variation in qPCR studies. 

According to our previous study in which hiPSC-CMs were cultured with a vascular-like network formed by human foreskin fibroblasts and human umbilical vein endothelial cells, the construct improved the hiPS-CM structure toward a more rod-like shape [25]. hiPSC-CMs had more elongated morphology and aligned with the tubular structures of the vascular-like network. Thus, our hypothesis in this present study was whether textile fibers would have similar orientating effect on the hiPSC-CMs as the tubular vascular structures. Our results support this hypothesis: Culturing of hiPSC-CMs on the PET textile had a positive effect on the morphology of the cells. 

Previous studies analyzing the effect of CM orientation and anisotropy on calcium handling of hiPSC CMs have reported inconsistent results [16,18,21,38,43,44]. According to our data, CMs cultured on PET textiles had slightly altered calcium handling properties, but no significant changes were observed. We also studied the adrenaline response of hiPSC-CMs. Adrenaline significantly increased beating frequency and decreased Ca^2+^ peak duration in CMs grown both on PET and on control coverslips. Adrenaline decreased the calcium transient decay time, indicating improved calcium reuptake. Therefore, the beta-adrenergic pathway is functional in the hiPS-CMs grown on PET 5 and the cells respond to adrenaline as expected.

Throughout the study, the attachment of the dissociated hiPS-CMs to the textile structure was poor. To improve the attachment, multiple coating materials were tested. Regardless of the coating material, the level of attachment remained the same. A portion of the cells slid through the textile fibers without attaching to the substrate. After PET textile removal from the cell culture well, a high number of vital hiPS-CMs was observed (data not shown). However, regardless of the low number of cells attached, the cells that were attached to the material remained viable for an extended period of time.

Culturing on PET textiles supports the formation of an oriented sarcomere structure as well as alignment of the hiPSC-CMs, thus inducing the structural maturation of these cells. However, the textile culturing had only minor effects on the expression levels of the cardiac-specific genes. Additionally, the functionality was comparable to the culturing on gelatin-coated glass surfaces. Notably, compared to the micro- and nano-grooved culture surfaces [18,41], one cross-sectionally round PET fiber had similar effects on the hiPS-CMs’ structure. It was earlier speculated that the edges of the grooves are especially important in the orientation of the cells. Here, we showed that the cross-sectionally round fibers of the textile structure support the cells and have a similar orientation effect on hiPS-CMs.

According to the results of the present study, for disease modeling studies as well as for drug screening and toxicology experiments, culturing of hiPS-CMs on textile structures would be beneficial. Compared to 2D cultures, culturing of hiPS-CMs would produce more standardized cultures, so the hiPS-CM population is more homogeneous in terms of cell structure and orientation. Therefore, for example, the effects of potential drug molecules on the cell structure and sarcomeres could be more reliably studied. However, more optimization is needed for the textile material, and the time scale for cell culturing on the scaffolds should be extended in future studies. Softer and more elastic textile material may be more suitable for hiPS-CMS. Material optimization is left for future studies.

## 5. Conclusions

In the present study, cardiomyocytes differentiated from the hiPSCs were cultured on PET textiles, and their structural properties, expression of the cardiac specific genes, and calcium handling properties were assessed. Based on the results, culturing hiPSC-CMs on the PET textiles improved their structural properties, such as elongation and sarcomere orientation, as well as improved the expression of the sarcomeric genes such as *TNNT2*. However, no statistically significant changes in the expression of the genes encoding cardiac ion channels or in the calcium handling properties of the hiPS-CMs were observed, and only minor changes were observed in their functionality as suggested by Ca^2+^ transients.

## Figures and Tables

**Table 1:** Details, manufacturers, and warp and weft type and diameter of a single filament (mean + SD) of the different PET textiles. Types 1, 2, and 3 were commercial textiles produced by Inka Oy, Killinkoski, Finland. Types 4 and 5 were produced at Tampere University, Tampere, Finland. Warp beams were provided by Finn-Nauha Oy, Haapamäki, Finland (yarn from Sinterama, Biella, Italy).

Textile Type and Details	Figure	Manufacturer	Warp/Weft	Single Filament ø (µm)
PET 1 Colorless, heat treated	1A	Inka Oy, Killinkoski, Finland	Textured/textured	24.4 ± 1.82
PET 2 Colorless, heat treated	1B	Inka Oy, Killinkoski, Finland	Straight/textured	23.2 ± 1.43
PET 3 Colorless, heat treated	1C	Inka Oy, Killinkoski, Finland	Textured/textured	22.9 ± 1.91
PET 4 Blue, heat treated	1D	Yarn: Finn-Nauha Oy, Haapamäki, Finland Textile: Tampere University of Technology	Straight/straight	20.4 ± 1.53
PET 5 Blue, heat treated	1E	Yarn: Finn-Nauha Oy, Haapamäki, Finland Textile: Tampere University of Technology	Straight/straight	22.0 ± 1.4

**Table 2:** TaqMan assays used in the Quantitative Reverse Transcription-Polymerase Chain Reaction (qRT-PCR).

Gene	Description	Function	TaqMan Assay ID
*MYL2*	Myosin regulatory light chain 2	Sarcomeric gene	Hs00166405_m1
*MYL7*	Myosin regulatory light chain 7	Sarcomeric gene	Hs01085598_g1
*MYL9*	Myosin regulatory light chain 9	Sarcomeric gene	Hs00697086_m1
*MYH6*	Myosin heavy chain 6	Sarcomeric gene	Hs01101425_m1
*MYH7*	Myosin heavy chain 7	Sarcomeric gene	Hs01110632_m1
*TNNC1*	Slow skeletal and cardiac type troponin C1	Sarcomeric gene	Hs00896999_g1
*TNNT2*	Cardiac type troponin T2	Sarcomeric gene	Hs00165960_m1
*ACTN2*	α-actinin 2	Sarcomeric gene	Hs00153809_m1
*TTN*	Titin	Sarcomeric gene	Hs00399225_m1
*MYBPC3*	Myosin binding protein C, cardiac	Sarcomeric gene	Hs00165232_m1
*TPM1*	α-tropomyosin	Sarcomeric gene	Hs00165966_m1
*KCNH2*	Potassium voltage-gated channel subfamily H member 2	Potassium channel	Hs04234270_g1
*KCNH6*	Potassium voltage-gated channel subfamily H member 6	Potassium channel	Hs00229215_m1
*KCNA10*	Potassium voltage-gated channel subfamily A member 10	Potassium channel	Hs1563550_s1
*KCND3*	Potassium voltage-gated channel subfamily D member 3	Potassium channel	Hs00542597_m1
*KCNQ1*	Potassium voltage-gated channel subfamily Q member 1	Potassium channel	Hs00923522_m1
*HCN4*	Hyperpolarization activated cyclic nucleotide-gated potassium channel 4	Potassium channel	Hs00975492_m1
*SCN5A*	Voltage-gated sodium channel, V type, alpha subunit	Sodium channel	Hs00165693_m1
*CACNA1C*	Voltage-dependent calcium channel, L type, alpha 1C subunit/CaCNA1.2	Calcium channel	Hs00167681_m1
*SLC8A1*	Solute carrier family 8, member 1/NCX1	Sodium-calcium exchanger	Hs01062258_m1
*PLN*	Phospholamban	Protein kinase substrate	Hs01848144_s1
*ATP2A2*	ATPase, calcium transporting, cardiac muscle, slow twitch 2/ SERCA2a	Calcium ATPase	Hs00544877_m1
*EEF1A1*; *EE*+	Eukaryotic translation elongation factor 1 alpha 1	Housekeeping gene	Hs00265885_g1
*GAPDH*	Glyceraldehyde-3-phosphate dehydrogenase	Housekeeping gene	Hs02758991­_g1
*TBP*	TATA-box binding protein	Housekeeping gene	Hs00427620_m1

**Table 3:** The data of the CytoSpectre analysis of cells grown on PET 5 and glass coverslips (control). The orientation of the CM sarcomeres was significantly higher on PET 5 compared to control (*p* < 0.05) as indicated by the average circular variance. The difference in sarcomere length between PET 5 and control samples was not significant. However, the shape of CMs was determined using the aspect ratio and CMs grown on PET 5 had a significantly higher ratio than the control (*p* < 0.05).

Sample	Average Circular Variance (0–1)	Average Modal Sarcomere Length (μm)	Average Aspect Ratio (Length to Width)	Number of Cells Analyzed
PET 5	0.611 ± 0.162	1.736 ± 0.187	4.915 ± 2.263	98
Control	0.882 ± 0.069	1.749 ± 0.122	1.567 ± 0.455	174

**Table 4:** Functionality of the hiPS-CMs cultured on PET 5 analyzed using Ca2+ imaging. The structure of the PET 5 textile did not hinder the Ca2+ imaging and the calcium handling properties were assessable from the hiPSC-CMs cultured on PET 5 textiles. Culturing on the PET 5 textiles slightly altered the calcium handling properties of the hiPSC-CMs; however, no significant changes were observed.

Sample	Peak Duration (ms)	Peak Amplitude (ΔF/F_0_)	Rise Time from 10% to 90% (ms)	Decay Time from 90% to 10% (ms)	Peak Frequency (Hz)	Cell Number
PET 5	582 ± 229	0.048 ± 0.037	112 ± 49	295 ± 131	0.93 ± 0.52	160
Control	590 ± 202	0.067 ± 0.030	90 ± 41	324 ± 96	0.75 ± 0.34	40

**Table 5:** Adrenaline significantly increased beating frequency and decreased peak duration in CMs grown on PET 5 textiles.

Sample	Peak Duration (ms)	Peak Amplitude (ΔF/F_0_)	Rise Time from 10% to 90% (ms)	Decay Time from 90% to 10% (ms)	Peak Frequency (Hz)
Baseline	648 ± 101	0.0360 ± 0.0183	115 ± 31	328 ± 73	0.709 ± 0.254
Adrenaline	614 ± 87	0.0310 ± 0.0139	122 ± 34	303 ± 63	0.859 ± 0.242

## Supplementary Materials

The following are available online at https://www.mdpi.com/1996-1944/12/11/1805/s1, Figure S1: Immunostaining of hiPS-CMs on different coating materials, Figure S2: The expression levels of the genes coding cardiac ion channels, Table S1: Data describing the structural maturation state of the hiPS-CMs with the tested coating materials.
