# Supplementary material for: Polyethylene Terephthalate Textiles Enhance the Structural Maturation of Human Induced Pluripotent Stem Cell-Derived Cardiomyocytes
Source: Materials (Basel). 2019 Jun 3;12(11):1805. doi: 10.3390/ma12111805 (PMC6600740; doi:10.3390/ma12111805)
Supplement: Supplementary file 1 [file materials-12-01805-s001.pdf]

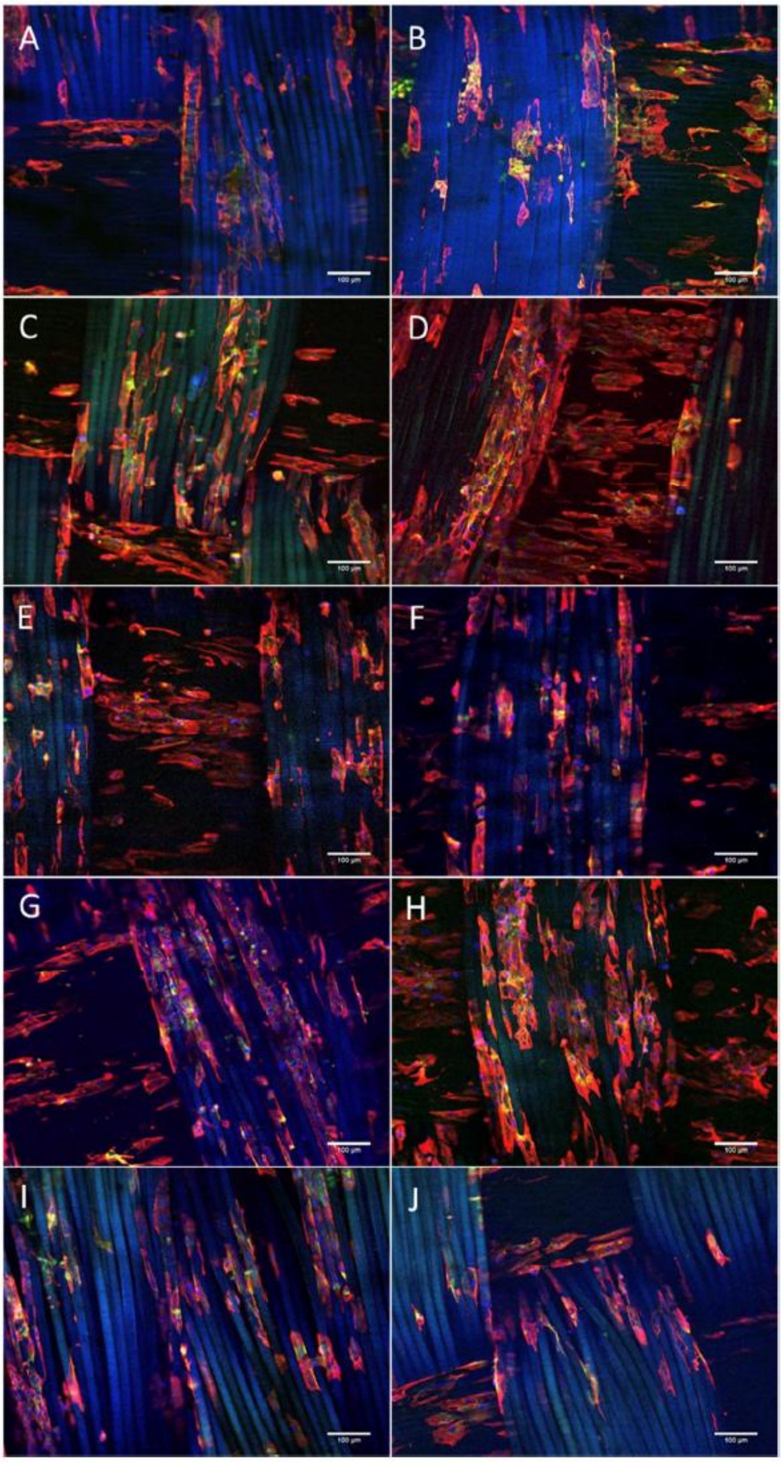

Figure S1. Representative images of the hiPS-CMs cultured on PET 5 samples coated using different coating materials, and immuno-stained with nuclei stain DAPI (blue), Myosin binding protein C (green), and cardiac Troponin T (red). The PET 5 fibers are seen in blue due to the autofluorescence. Five different surface coatings for PET 5 samples were used: (**A**,**B**) cells plated on dopamine-bound gelatin coating, (**C**,**D**) cells plated on gelatin coating, (**E**,**F**) cells plated on Geltrex™ coating, (**G**,**H**) cells plated on plasma-treated textile without any coating, and (**I**,**J**) cells plated on PET that was plasma-treated and gelatin-coated. According to the visual inspection, no significant differences were found between the coatings. There are hiPS-CMs attached on the fibers of PET textile and they were oriented according to the fibers regardless of the coating material. The other coatings were not superior to the original gelatin coating. The scale bars represent 100 μm.

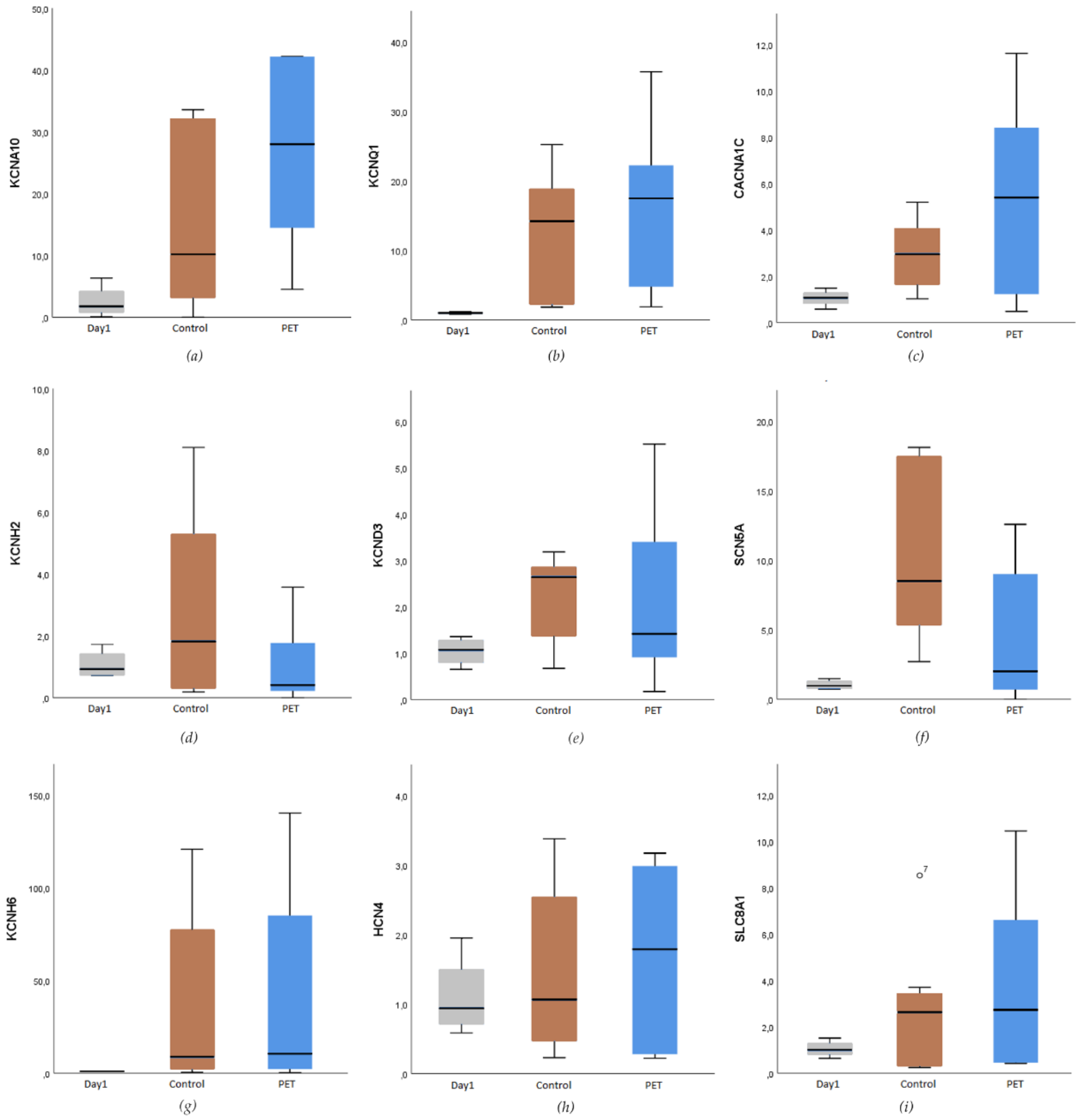

Figure S2. The expression levels of the genes coding for cardiac ion channels on hiPS-CMs cultured on PET 5 and control surfaces (glass coverslips) compared to the day one sample. (**a**) Potassium voltage-gated channel subfamily A member 10 (*KCNA*10), (**b**) potassium voltage-gated channel subfamily Q member 1 (*KCNQ1*), (**c**) voltage-dependent calcium channel, L type, alpha 1C subunit/CaCNA1.2 (*CACNA1C*), (**d**) potassium voltage-gated channel subfamily H member 2 (*KCNH2*), (**e**) potassium voltage-gated channel subfamily D member 3 (*KCND3*), (**f**) voltage-gated sodium channel, V type, alpha subunit (*SCN5A*), (**g**) potassium voltage-gated channel subfamily H member 6 (*KCNH6),* (**h**) hyperpolarization activated cyclic nucleotide-gated potassium channel 4 (*HCN4*), and (**i**) solute carrier family 8, member 1/NCX1 (*SLC8A1*). The genes were expressed on both surfaces in a similar manner and no significant differences were seen.

Table S1. The structural maturation state of the hiPS-CMs with the tested coating materials. There were no significant differences found between different coating materials.

**Coating material**	**Average circular variance (0–1)**	**Average modal sarcomere length (µm)**	**Average aspect ratio (length to width)**	**Number of nuclei per mm^2 (mean ± SD) *n* = 5–6 images from 2 independent samples**
Geltrex™ (*n* = 22)	0.349 ± 0.139	1.56 ± 0.13	3.53 ± 1.27	223 ± 87
Gelatin (*n* = 14)	0.363 ± 0.098	1.64± 0.25	3.71 ± 1.07	260 ± 130

Plasma treatment prior gelatin coating (n = 54)	0.387 ± 0.149	1.68± 0.16	3.74 ± 1.77	283 ± 60
